# Supplementary material for: Cell Penetrating Peptide as a High Safety Anti-Inflammation Ingredient for Cosmetic Applications
Source: Biomolecules. 2020 Jan 7;10(1):101. doi: 10.3390/biom10010101 (PMC7023394; doi:10.3390/biom10010101)
Supplement: Supplementary file 1 [file biomolecules-10-00101-s001.pdf]

biomolecules

Supplementary Materials

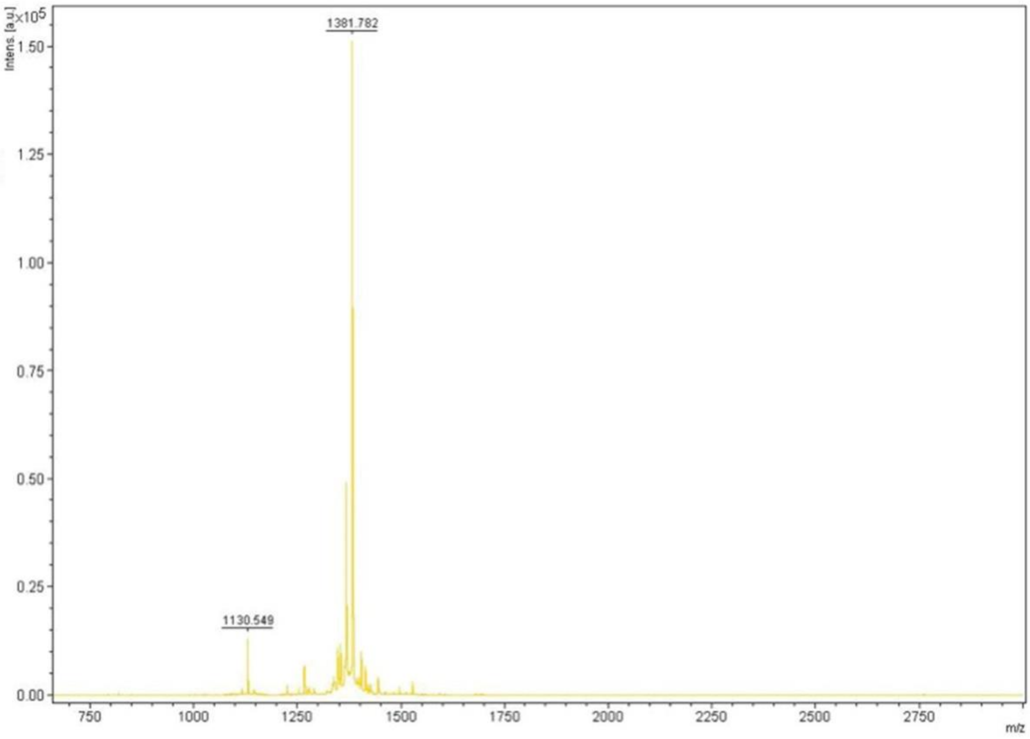

Figure S1 MALDI-TOF mass profile of CPP_{AIF}.

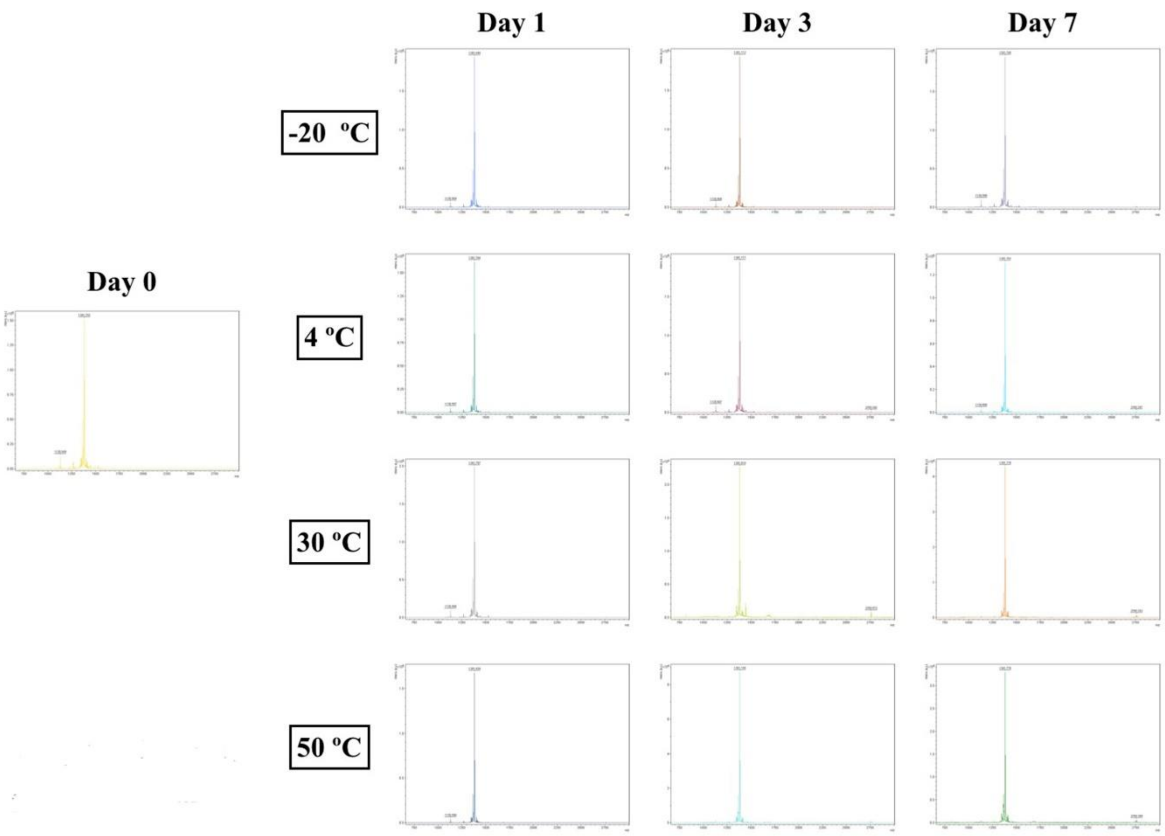

Figure S2 MALDI-TOF mass profile of CPP_{AIF} in water under different temperature for 1, 3 and 7 days. CPP_{AIF} was dissolved in water at various temperature (-20 °C, 4 °C, 30 °C, 50 °C) for different duration and characterized by mass spectrometry.

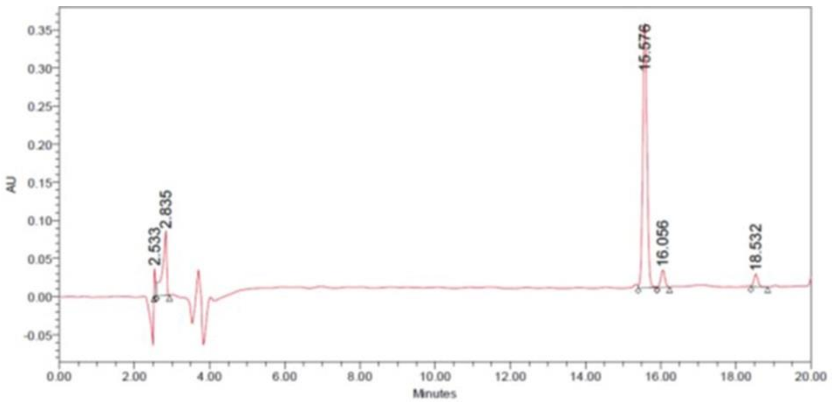
0.35
0.30
0.25
0.20
0.15
0.10
0.05
0.00
-0.05
AU
2.533
2.835
15.576
16.056
18.532
0.00
2.00
4.00
6.00
8.00
10.00
12.00
14.00
16.00
18.00
20.00
Minutes

(A)

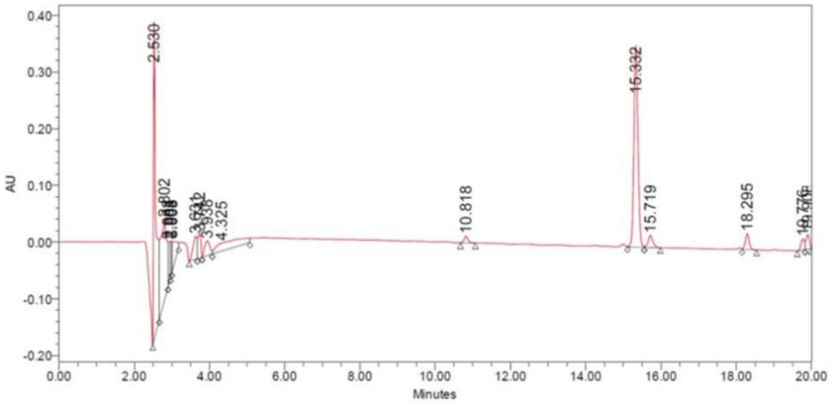
0.40
0.30
0.20
0.10
0.00
-0.10
-0.20
AU
2.530
3.631
4.325
10.818
15.332
15.719
18.295
0.00
2.00
4.00
6.00
8.00
10.00
12.00
14.00
16.00
18.00
20.00
Minutes

(B)

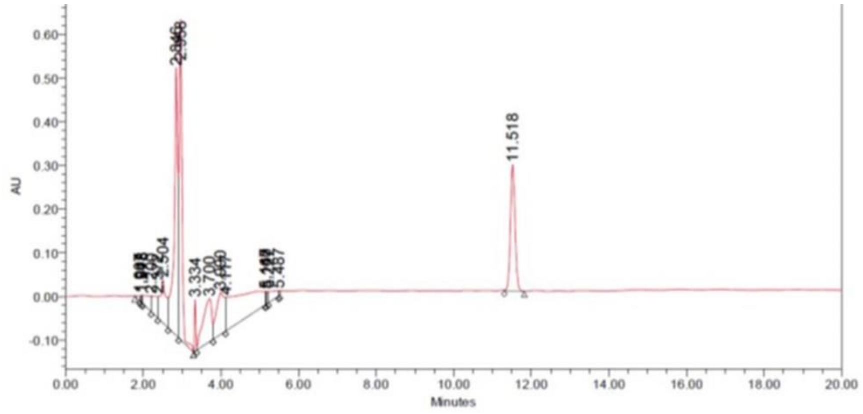

(C)

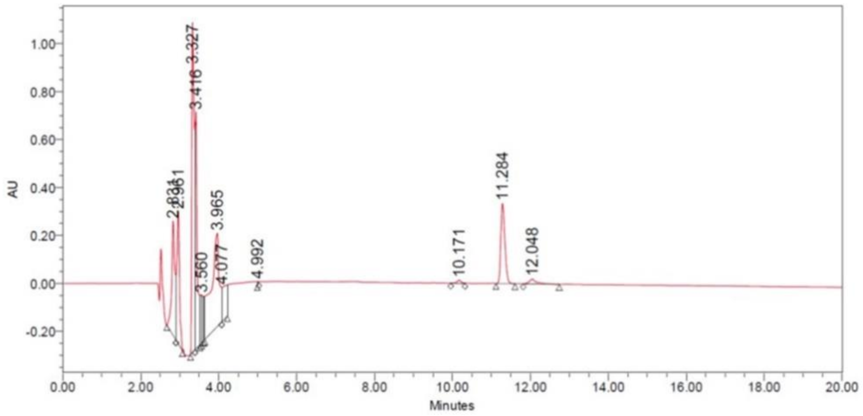

(D)

Figure S3 The HPLC chromatograms for cysteine and lysine depletion quantification of CPP$_{AIF}$. (A) cysteine only; (B) CPP$_{AIF}$ with cysteine; (C) lysine only; (D) CPP$_{AIF}$ with lysine.

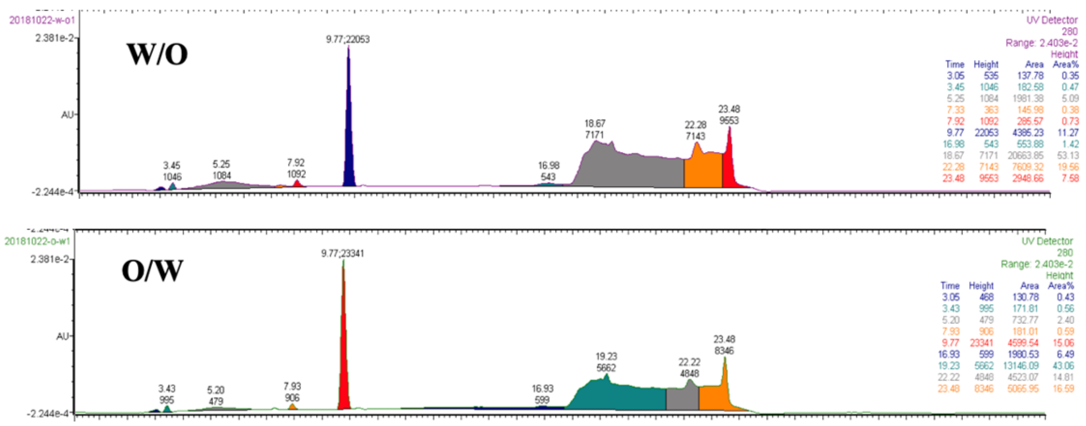

Figure S4 HPLC chromatogram of CPP_{AIF} in different formulations applied in reconstructed human epidermis tissue model. W/O: CPP_{AIF} in W/O formulation; O/W: CPP_{AIF} in O/W formulation.

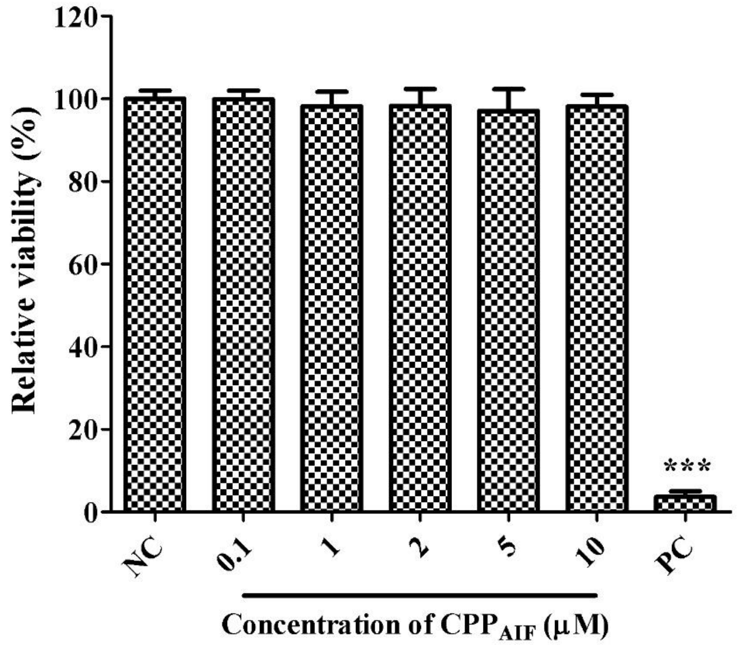

Figure S5 Cytotoxicity test of CPPAIF to HaCaT cells. HaCaT cells (1x103) were seeded in 96-well plate and incubated at 37 °C for 16 h. The cells were then incubated different concentrations of CPPAIF for 2 h. The cells were washed with PBS and cell viability was measured by AlamarBlue cell viability assay. PBS was applied as negative control (NC) set as 100% (mock) and 5% SDS was applied as positive control (PC). *** $p < 0.001$ versus the NC.

Table S1 O/W formulation content

Trade Name	INCI Name	%
Aqua	Aqua	qsp 100
Glycerine 4812	Glycerin	10
Purolan PE	Phenoxyethanol	0.8
Dermosoft® Octiol	Caprylyl Glycol	0.2
Mihacol 139	Sodium Acrylate/Sodium Acryloyldimethyl Taurate Copolymer, Mineral Oil (Paraffinum Liquidum)& Trideceth-6	1
Cordamol GTIS	Triisostearin	10

Table S2 W/O formulation content

Trade Name	INCI Name	%
Aqua	Aqua	qsp 100
Glycerine 4812	Glycerin	10
Purolan PE	Phenoxyethanol	0.8
Dermosoft® Octiol	Caprylyl Glycol	0.2
NaCl	Sodium Chloride	1
Dow Coring® ES-5300 formulation Aid	Lauryl PEG-10 Tris (trimethylsiloxy) silylethyl Dimethicon	2
KF-995	Cyclopentasiloxane	20
BF-1169	C13-16 Isoparaffin, Dimethicone	5

© 2020 by the authors. Submitted for possible open access publication under the terms and conditions of the Creative Commons Attribution (CC BY) license (http://creativecommons.org/licenses/by/4.0/).
